# Risk Assessment of Bone Metastasis for Cervical Cancer Patients by Multiple Models: A Large Population Based Real-World Study

**DOI:** 10.3389/fmed.2021.725298

**Published:** 2021-10-05

**Authors:** Yun Han, Bo Wang, Jinjin Zhang, Su Zhou, Jun Dai, Meng Wu, Yan Li, Shixuan Wang

**Affiliations:** ^1^National Clinical Research Center for Obstetrical and Gynecological Diseases, Wuhan, China; ^2^Key Laboratory of Cancer Invasion and Metastasis, Ministry of Education, Wuhan, China; ^3^Department of Obstetrics and Gynecology, Tongji Hospital, Tongji Medical College, Huazhong University of Science and Technology, Wuhan, China

**Keywords:** cervical cancer, bone metastasis, predictive model, machine learning algorithm, SEER

## Abstract

**Background:** Population-based data on the risk assessment of newly diagnosed cervical cancer patients' bone metastasis (CCBM) are lacking. This study aimed to develop various predictive models to assess the risk of bone metastasis via machine learning algorithms.

**Materials and Methods:** We retrospectively reviewed the CCBM patients from the Surveillance, Epidemiology, and End Results (SEER) database of the National Cancer Institute to risk factors of the presence of bone metastasis. Clinical usefulness was assessed by Akaike information criteria (AIC) and multiple machine learning algorithms based predictive models. Concordance index (C-index) and receiver operating characteristic (ROC) curve were used to define the predictive and discriminatory capacity of predictive models.

**Results:** A total of 16 candidate variables were included to develop predictive models for bone metastasis by machine learning. The areas under the ROC curve (AUCs) of the random forest model (RF), generalized linear model (GL), support vector machine (SVM), eXtreme Gradient Boosting (XGBoost), artificial neutral network (ANN), decision tree (DT), and naive bayesian model (NBM) ranged from 0.85 to 0.93. The RF model with 10 variables was developed as the optimal predictive model. The weight of variables indicated the top seven factors were organ-site metastasis (liver, brain, and lung), TNM stage and age.

**Conclusions:** Multiple machine learning based predictive models were developed to identify risk of bone metastasis in cervical cancer patients. By incorporating clinical characteristics and other candidate variables showed robust risk stratification for CCBM patients, and the RF predictive model performed best among these predictive models.

## Introduction

Cervical cancer is one of the most common and deadly cancers in low-income and middle-income countries. Each year, more than half a million women are diagnosed with cervical cancer and the disease results in over 300,000 deaths worldwide (1). To date, multimodal therapy is promising for early-stage or locally advanced cervical cancer patients. However, there is no specific widely accepted access for cervical cancer patients with metastasis because of heterogeneous manifestations (2).

According to the International Federation of Gynecology and Obstetrics (FIGO) stage criteria, cervical cancer patients with FIGO stage I to IV, or according to the American Joint Committee Cancer (AJCC) criteria, any AJCC tumor[T] stage, lymph node[N] stage, and distant metastasis of peritoneal spread and involvement of supraclavicular-, para-aortic-, or mediastinal lymph node [M1], organ-site metastasis (lung, liver, brain, or bone) at initial diagnosis, or who have persistent/recurrent disease outside the pelvis, are classified as metastatic cases (3). For patients presenting with isolated or multiple metastasis who received more than one prior systemic therapy had dismal outcomes (4). Collectively, the survival outcomes of patients with metastatic cervical cancer are poor.

Bone is the third most common site of distant metastasis after the lung and liver (5). The incidence of bone metastasis from the carcinoma of the uterine cervix were reported from 0.8 to 23% (5–8). For most of CCBM patients, lesions of the bone were detected within 1 year after completion of the initial treatments by bone scan, FDG-PET, X-ray, or MRI (6). In short, there is a lag in diagnosis which make severe influence in prognosis. So early prediction of the occurrence of bone metastases and immediate treatment is important, to improve the quality of life in patients with cervical cancer. Besides, there is no standard accepted guideline for the treatment of CCBM patients involvement because of its low prevalence and the lack of large population-based study. Consequently, there is an urgent need to develop an accurate model for predicting the risk and survival outcome of CCBM patients that can be used to facilitate the management of clinical treatment.

In the present study, we established multiple predictive models which use data classification algorithm, including generalized linear model, random forest, support vector machine, extreme gradient boosting, artificial neutral network, decision tree, naive bayesian model, based on supervised machine learning algorithm to predict the risk factors for CCBM patients using the Surveillance, Epidemiology, and End Results (SEER) database. We then analyzed the predictive performance of this nomogram in a deviation cohort and then verified performance in an internal validation cohort.

## Materials and Methods

### Patients Enrollment From the SEER Database

**Figure 1:**
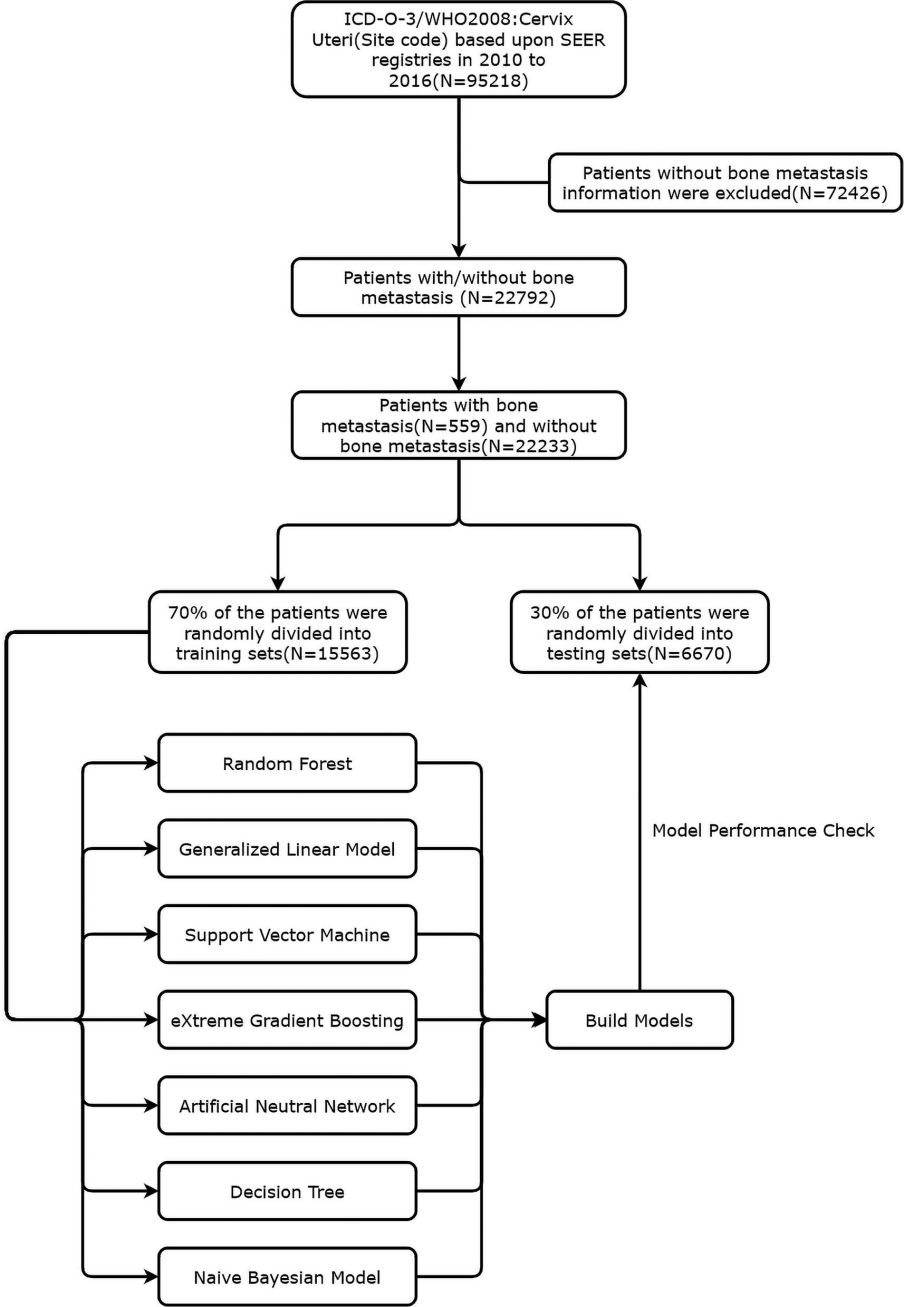
Flow chart of cervical patients inclusion and model establishment.

Between January 1, 2010 and December 31, 2016, we retrospectively collated data from consecutive patients who had been diagnosed with cervical cancer from the SEER database. Data were acquired to generate the case listing via the SEER^*^Stat software version 8.3.6 (https://seer.cancer.gov/data/). Since the SEER data are anonymized, the need for institutional review board approval was waived. The SEER 18 registries were used for cases selection, which representing ~30% of the US population (9). According to the International Classification of Diseases for Oncology-3 (ICD-O-3)/WHO 2008, the entry name is “cervix uteri.” The exclusion criteria were as follows: (1) patients for whom the presence or absence of bone metastasis at diagnosis was unknown; (2) patients diagnosed at autopsy or death certificates; (3) patients younger than 18 years old; (4) patients diagnosed with carcinoma *in situ*, benign or borderline tumors. Besides, for individual patient IDs with multiple records, the primary registry was included. Hence, derived AJCC 6th and SEER combined stage (2016+) were used for tumor node metastasis (TNM) staging classification in our study. Figure 1 presented a flowchart of data screening from the SEER database and subsequent analysis followed.

### Study Covariables

We collected demographical and clinical variables as follows: age at initial diagnosis, race [White, Black, and other (American Indian/Alaska Native, Asian Native, and Asian/Pacific Islander)], the year of diagnosis, primary site, the SEER historic, lymph biopsy, regional lymph nodes examined, surgery, tumor size, marital status, tumor grade [well-differentiated (grade1), moderately differentiated (grade2), poorly differentiated (grade 3), pathology, and undifferentiated (grade 4)], survival status, survival time [median (IQR)], distant lymph metastasis and the presence of other distant site metastasis (brain, liver and lung), TNM staging (Tumor, Node, and metastasis), insurance status.

### Construction of Machine Learning Based Predictive Models

According to the rules of clinical predictive model establishment, all CCBM patients were randomly divided into training set and test set by 7:3, keeping the distribution of bone metastasis data in both groups consistent. Seven supervised learning model were developed to predict the risk of bone metastasis, including random forest model (RF), generalized linear model (GL), support vector machine (SVM), eXtreme Gradient Boosting (XGBoost), artificial neutral network (ANN), decision tree (DT), and naive bayesian model (NBM).

### Strategy for Eigenfactor Selection and Model Validation

In order to avoid over fitting the model and the loss of information as much as possible, the EasyEnsemble, BalanceCascade and 10-fold cross-validation were used to select eigenfactor. For each repeated time, subsets were randomly arranged in the training and test group. The rank of each candidate variable from the training set was included in the seven machine learning based predictive model, and validated in the test set.

### Statistical Analysis

Continuous variables are expressed as mean (standard deviation) and compared using the two-tailed *t*-test or the Mann-Whitney test. Categorical variables were compared using the χ^2^ test or Fisher's exact test. To explore potential predictive factors, we also calculated the odds ratio (OR) and the corresponding 95% confidence interval (CI) from the generalized linear (GLM) model. The risk factors for cervical cancer patients with bone metastasis were predicted primarily by univariable logistic regression. The useful univariable logistic regression (*P* < 0.05) were considered as candidates for the further multivariable logistic analysis. A nomogram was formulated based on results arising from the Akaike information criteria (AIC) analysis. The nomogram was based on the proportional conversion of each regression coefficient in the multivariate logistic regression to a 0 to 100-point scale. The effect of the variable with the highest β coefficient (the absolute value) was assigned 100 points (10). Points were added for all independent variables in order to create a total which was then converted to predicted probabilities. Next, we used bootstrapping plots to calculate the concordance index (C-index) and area under the receiver operating characteristic curve (AUC) so that we could evaluate our ability to calibrate the curve. Typically, C-index and AUC values that exceeded 0.6 were suggestive of a reasonable estimation. We also used net reclassification index (NRI) and integrated discrimination improvement (IDI) to evaluate the clinical benefits and utility of the nomogram, as described previously (11, 12). The cut-off point for risk stratifications was selected using X-tile. All analyses were conducted using SAS, version 9.1 (SAS Institute Inc.) and the R statistical package (v.3.6.2; R Foundation for Statistical Computing, Vienna, Austria; https://www.r-project.org). A *P*-value < 0.05 was considered to be statistically significant.

## Results

### Patient Characteristics

A total of 22,792 of CCBM patients' clinical characteristics and pathological baseline data were summarized in Table 1. The old patients (age ≥50) presented with a significantly increased incidence of bone metastasis compared with patients with young age (*P* < 0.001). Moreover, patients with high grade, pathology (adenocarcinoma vs. squamous cell carcinoma), lymph vascular invasion (diagnosed 2010+ for the schemas for penis and testis only), TNM stage, lymph biopsy (regional lymph nodes removed or not), surgery, regional lymph nodes examination, distant site metastasis (liver, brain and lung), and tumor size also contributed to higher bone metastasis incidence. We constructed generalized linear model, random forest model and another five supervised machine learning algorithm in classification outcomes predication. Besides, to develop machine learning based predictive models, a total of 16 features were selected: age at initial diagnosis (as continuous variable), race, primary site (Cervix uteri, Endocervix, Exocervix equivalent FIGO I, Overlapping lesion of cervix uteri equivalent FIGO II), the SEER historic, surgery, tumor size, distant lymph metastasis, tumor grade, pathology and the presence of other distant site metastasis (brain, liver and lung), TNM staging (Tumor, Node, and metastasis), insurance status. The whole patients were randomly split into a training set (*N* = 15,954, 70%) and validation set (*N* = 6,838, 30%).

**Table 1:** Baseline demographic and clinical characteristics of included patients diagnosed with and without bone metastasis.

**Variables**	**Level**	**Bone metastasis**		***P*-value**
		**Yes** **(*N* = 559)**	**No** **(*N* = 22,233)**	
Age [median (IQR)]		56.00 [47.00, 65.00]	49.00 [39.00, 61.00]	<0.001
**Race (%)**	Black	97 (17.4)	3,063 (13.8)	0.006
	Other	52 (9.3)	2,355 (10.6)	
	Unknown	0 (0.0)	253 (1.1)	
	White	410 (73.3)	16,562 (74.5)	
**Year (%)**	2010	60 (10.7)	3,167 (14.2)	0.004
	2011	60 (10.7)	3,137 (14.1)	
	2012	74 (13.2)	3,174 (14.3)	
	2013	82 (14.7)	3,001 (13.5)	
	2014	88 (15.7)	3,226 (14.5)	
	2015	108 (19.3)	3,252 (14.6)	
	2016	87 (15.6)	3,276 (14.7)	
Primary_site^*^ **(%)**	Cervix uteri	495 (88.6)	17,208 (77.4)	<0.001
	Endocervix	56 (10.0)	4,267 (19.2)	
	Exocervix	2 (0.4)	410 (1.8)	
	OLC	6 (1.1)	348 (1.6)	
**Grade (%)**	Grade I	10 (1.8)	2,527 (11.4)	<0.001
	Grade II	89 (15.9)	6,946 (31.2)	
	Grade III	229 (41.0)	6,202 (27.9)	
	Grade IV	27 (4.8)	516 (2.3)	
	Unknown	204 (36.5)	6,042 (27.2)	
**Pathology (%)**	ADC	78 (14.0)	4,202 (18.9)	<0.001
	SCC	314 (56.2)	14,253 (64.1)	
	Others	167 (29.9)	3,778 (17.0)	
**SEER_historic**^**ϵ**^ **(%)**	Distant	472 (84.4)	2,363 (10.6)	<0.001
	Localized	0 (0.0)	8,787 (39.5)	
	Regional	0 (0.0)	7,427 (33.4)	
	Unknown	87 (15.6)	3,656 (16.4)	
**T_stage**^** 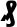 **^ **(%)**	T0	2 (0.4)	11 (0.0)	<0.001
	T1	62 (11.1)	10,401 (46.8)	
	T2	83 (14.8)	4,163 (18.7)	
	T3	186 (33.3)	2,865 (12.9)	
	T4	57 (10.2)	676 (3.0)	
	TX	78 (14.0)	691 (3.1)	
	Unknown	91 (16.3)	3,426 (15.4)	
**N_stage**^** 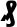 **^ **(%)**	N0	119 (21.3)	13,562 (61.0)	<0.001
	N1	291 (52.1)	4,552 (20.5)	
	NX	58 (10.4)	693 (3.1)	
	Unknown	91 (16.3)	3,426 (15.4)	
**M_stage**^** 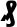 **^ **(%)**	M0	0 (0.0)	16,641 (74.8)	<0.001
	M1	468 (83.7)	2,166 (9.7)	
	Unknown	91 (16.3)	3,426 (15.4)	
**Lymph_biopsy (%)**	>4	8 (1.4)	7,258 (32.6)	<0.001
	≤ 3	7 (1.3)	458 (2.1)	
	Unknown	544 (97.3)	14,517 (65.3)	
**Surgery (%)**	No	520 (93.0)	9,654 (43.4)	<0.001
	Unknown	0 (0.0)	35 (0.2)	
	Yes	39 (7.0)	12,544 (56.4)	
**Regional_nodes_** **examined (%)**	Negative	512 (91.6)	13,787 (62.0)	<0.001
	Positive	28 (5.0)	8,138 (36.6)	
	Unknown	19 (3.4)	308 (1.4)	
**Bone_metastasis (%)**	No	0 (0.0)	22,233 (100.0)	<0.001
	Yes	559 (100.0)	0 (0.0)	
**Brain_metastasis (%)**	No	504 (90.2)	22,171 (99.7)	<0.001
	Unknown	20 (3.6)	12 (0.1)	
	Yes	35 (6.3)	50 (0.2)	
**Liver_metastasis (%)**	No	387 (69.2)	21,874 (98.4)	<0.001
	Unknown	14 (2.5)	25 (0.1)	
	Yes	158 (28.3)	334 (1.5)	
**Lung_metastasis (%)**	No	314 (56.2)	21,435 (96.4)	<0.001
	Unknown	22 (3.9)	48 (0.2)	
	Yes	223 (39.9)	750 (3.4)	
**Distant_Lymph_** **metastasis (%)**	No	44 (7.9)	3,033 (13.6)	<0.001
	Unknown	474 (84.8)	18,969 (85.3)	
	Yes	41 (7.3)	231 (1.0)	
**CS_tumor_size (%), cm**	<5	251 (44.9)	13,007 (58.5)	<0.001
	≥5	1 (0.2)	454 (2.0)	
	Unknown	307 (54.9)	8,772 (39.5)	
**Insurance (%)**	Any Medicaid	183 (32.7)	6,704 (30.2)	0.306
	Insured	323 (57.8)	13,484 (60.6)	
	Uninsured	40 (7.2)	1,376 (6.2)	
	Unknown	13 (2.3)	669 (3.0)	
**Marital_status (%)**	Married	360 (64.4)	14,009 (63.0)	0.139
	Unknown	24 (4.3)	1,414 (6.4)	
	Unmarried	175 (31.3)	6,810 (30.6)	
**Survival status (%)**	Alive	117 (20.9)	16,507 (74.2)	<0.001
	Dead	442 (79.1)	5,726 (25.8)	
**Survival time [median (IQR)]**		5.00 [2.00, 12.00]	23.00 [9.00, 47.00]	<0.001

* *According to the primary site labeled. 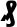
According to the American Joint Committee on Cancer (AJCC), 6th. € According to the SEER historic stage (1973–2015). IQR, Interquartile range; OLC, Overlapping lesion of cervix uteri; ADC, Adenocarcinoma; SCC, Squamous cell carcinoma*.

### Risk Assessment of Bone Metastasis With GL Model

**Figure 2:**
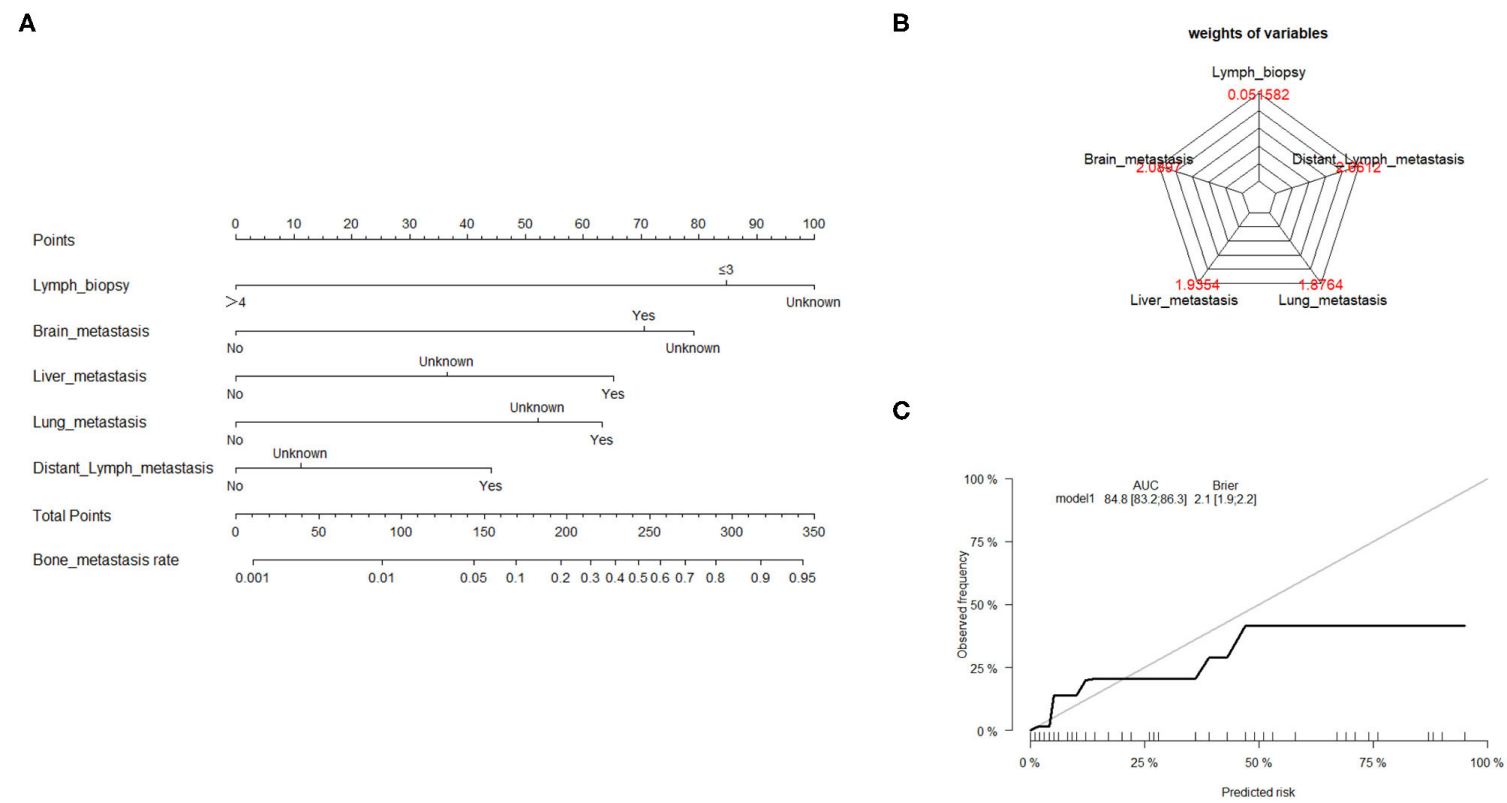
Nomogram to estimate the risk of bone metastasis. **(A)** A nomogram for predicting the risk of bone metastasis showing the proportion (%) of parameters included in the score scale. To use the bone metastasis nomogram score, it is important to identify the point of each variable on the corresponding axis; the total number of points can then be summated from all variables. **(B)** Radar plot showing the relative weight of candidate parameters arising from stepwise regression analysis. **(C)** Calibration curves depicting the robust performance of the nomogram in terms of consensus between the predicted risk and actual risk assessment.

Traditionally, linear regression has been the technique of choice for predicting medical risk (13). The GL model is reasonably well-known, with the exception of logistic, log-linear, and some survival models. The risk factors associated with bone metastasis were screened using univariate and multivariate logistic regression, as presented in Table 2. Based on the AIC results, the lymph biopsy, brain metastasis, liver metastasis, lung metastasis, and distant lymph metastasis were positively correlated with the development of bone metastasis. The nomogram was constructed using these five significant risk factors listed above (Figure 2A). The Brier score showed the robust accuracy of probabilistic predictions (Figure 2B). The C-indexes of the nomogram for predicting risk of bone metastasis were 0.85 (95% CI: 0.83–0.86), which also showed good predictive value of the nomogram in the validation cohort (Figure 2C).

**Table 2:** Multivariate logistic regression analysis for risk factors associated with bone metastasis in cervical patients.

**Variables**	**OR**	**95% CI**	***P*-value**
Age*	0.99	0.99, 1.00	0.11
**Race**			
Black	—	—	
Other	0.95	0.64, 1.40	0.8
Unknown	0	0, 0	>0.9
White	1.08	0.85	0.5
**Primary_site**			
Cervix uteri	—	—	
Endocervix	0.74	0.53, 1.02	0.076
Exocervix	0.3	0.05, 0.99	0.1
Overlapping lesion of cervix uteri	0.67	0.25, 1.51	0.4
**Grade**			
Gradel	—	—	
Gradell	1.42	0.74, 3.00	0.3
Gradelll	1.95	1.04, 4.07	0.051
GradeIV	1.74	0.81, 4.01	0.2
Unknown	1.77	0.95, 3.70	0.1
**Pathology**			
ADC	—	—	
Others	1.11	0.81, 1.55	0.5
SCC	0.89	0.66, 1.21	0.4
**SEER_historic**			
Distant	—	—	
Localized	0	0, 0	>0.9
Regional	0	0, 0	>0.9
Unknown	0.03	0, 0.1	<0.001
**Lymph_biopsy**			
>4	—	—	
≤ 3	5.03	1.70, 14.6	0.003
Unknown	9.22	4.82, 20.5	<0.001
**Brain_metastasis**			<0.001
No	—	—	
Unknown	8.85	3.42, 25.0	<0.001
Yes	3.28	2.02, 5.29	<0.001
**Liver_metastasis**			
No	—	—	
Unknown	2.28	0.86, 5.72	0.085
Yes	2.66	2.10, 3.37	<0.001
**Lung_metastasis**			
No	—	—	
Unknown	1.77	0.85, 3.52	0.11
Yes	1.6	1.30, 1.97	<0.001
**Distant_Lymph_metastasis**			
No	—	—	
Unknown	0.02	0.03, 0.67	0.029
Yes	6.18	3.87, 9.84	<0.001
**CS_tumor_size,cm**			
<5	—	—	
≥5	0.84	0.04, 5.14	0.9
Unknown	0.98	0.79, 1.21	0.8

^*^Continuous variable. ADC, Adenocarcinoma; SCC, Squamous cell carcinoma.

### Prediction of Bone Metastasis With RF Model

**Figure 3:**
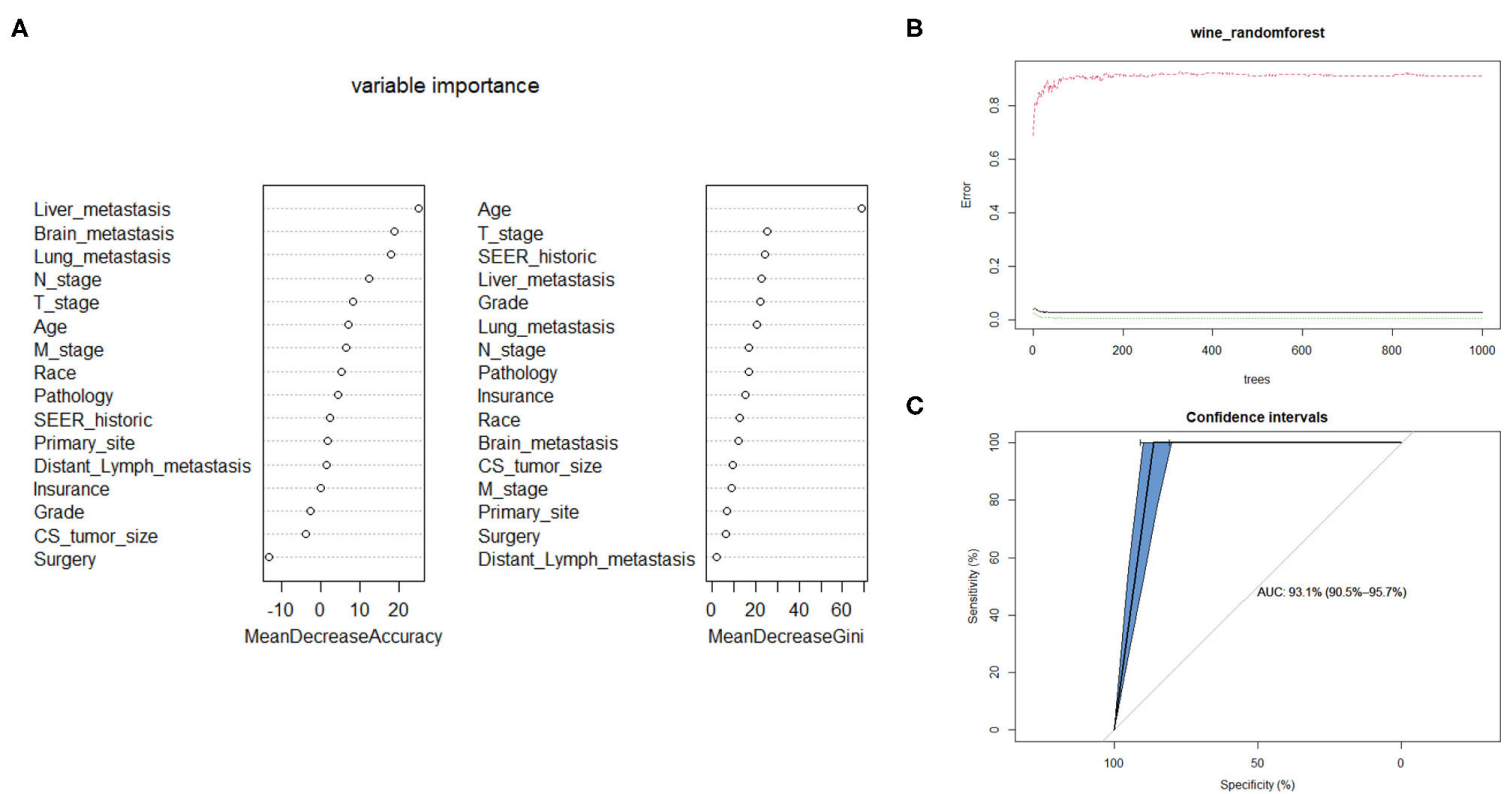
Random forest model. **(A)** The candidate factors associated with micrometastasis of lymph nodes were ordered according to the mean decreased Gini index. **(B)** Relationship of dynamic changes between the prediction error and the number of decision trees. **(C)** Performance of the prediction model with increasing numbers of features in the ROC curve.

The Random Forest technique has great advantages over other algorithms and performs well on many current data sets. It is a regression tree technique which uses bootstrap aggregation and randomization of predictors to achieve a high degree of predictive accuracy (14). Although random forest model cannot generate a score sheet, it can handle data of very high dimensions (many features) and give out which features are more important after training. In the forest, the class predictions produced by each tree were assembled and the model prediction was finally determined according to the majority vote (15). As indicated in Table 3, sixteen variables were ordered according to the Mean Decrease Gini index. The random forest could better distinguish cervical cancer patients with bone metastasis or not when the number of decision tree was 500 (Figure 3B). The AUC was 0.93 (95% CI: 0.91–0.96), which showed robust consistency between the probability and observation in the RF model (Figure 3C).

**Table 3:** The candidate variables screening associated with bone metastasis based on random forest model.

**Variables**	**Mean decrease accuracy**	**Mean decrease Gini**
Age*	0.000642813	68.83203688
Race	0.000274563	12.4840761
Grade	−0.000203516	21.95036003
Primary_site	6.99E-05	6.768598158
Pathology	0.00028804	16.5846293
SEER_historic	0.000485735	24.30262207
T_stage	0.001903695	24.99986783
N_stage	0.003697878	16.91665064
M_stage	0.002626603	8.880804155
Surgery	−0.000949919	6.36547638
Brain_metastasis	0.000877874	11.79487391
Liver_metastasis	0.003425153	22.55114629
Lung_metastasis	0.002344084	20.40700772
Distant_Lymph_metastasis	7.86E-05	1.938150108
CS_tumor_size	−0.000214407	9.573248324
Insurance	−2.43E-06	15.22568758

^*^Continuous variable. Pathology: Squamous cell carcinoma, adenocarcinoma, others. Primary_site: According to the primary site labeled (Cervix uteri, Endocervix, Exocervix, and Overlapping lesion of cervix uteri). TNM stage: tumor node metastasis classification (AJCC6th). AJCC, American Joint Committee on Cancer.

### Another Five Supervised Learning Models Developed for CCBM

On the basis of monofactor analysis of baseline characteristics of included patients, we further use another five supervised learning models to conduct CCBM risk assessment to see if we can improve prediction performance. A total of 16 candidate variables were used to develop predictive model for bone metastasis based on supervised learning algorithms. The predictive performance of all models were shown in Table 4. By feature selection, the variables for each algorithm were ranked by their predictive importance, the optimal permutation and combination of variables were included in model construction. The RF model with 10 variables, as shown in Figure 3A, had the highest net benefits almost across the entire range of threshold probabilities. Five models (SVM, XGBoost, ANN, DT, NBM) performed significantly better than the GL model at most of threshold points. Among these five model, we can see naive bayesian model is the best which has highest mean AUC.

**Table 4:** The predictive performance of candidate models based on maching learning algorithm.

**Model**	**AUC**		**No. of optimal variables**
	**Mean**	**95% CI**	
RF	0.93	0.91–0.96	10
GLM	0.85	0.83–0.86	5
SVM	0.89	0.87–0.91	7
XGBoost	0.88	0.85–0.91	10
ANN	0.91	0.88–0.94	10
DT	0.88	0.84–0.92	12
NBM	0.92	0.88–0.96	11

*RF, Random Forest; GLM, Generalized Linear Model; SVM, Support Vector Machine; XGBoost, eXtreme Gradient Boosting; ANN, Artificial Neutral Network; DT, Decision Tree; NBM, Naive Bayesian Model; AUC, Area under the curve*.

## Discussion

Hematogenous metastasis and lymphatic metastasis remain a major cause of cervical cancer related death in women (3). However, the bone manifestation is rare in cervical cancer patients. The rates of bone metastasis in cervical cancer patients with early-stage and advanced stage were reported from 4.0 to 22.9% (16–19). As for bone metastasis, vertebral column is the most frequent site, particularly the lumbar spine (20). Among the 22,792 patients with solitary metastasis or multiple metastasis analyzed for incidence, we found in this study that the incidence of cervical cancer with bone metastasis was 2.5%, consistent with previous studies (16, 21, 22). Early diagnosis and proper treatment of CCBM patients can prevent or relieve symptoms such as severe pain, pathological fracture, and even disability.

Currently, there are no referential screening guidelines for the warning of CCBM patients, the identification of predictive model for the development of bone metastasis could contribute to cervical cancer patients with high risk for developing bone metastasis, and if possible, a predictive model is guidable for appropriate preventive treatment at an early stage. In this study, we found that cervical cancer patients with older age (≥50 years), poorly differentiation, advanced stage, non-squamous histology type, combined with other organ metastasis and without operation at initial treatment were more inclined to suffering bone metastasis. Indeed, it's not hesitated that cervical cancer patients with elder age, advanced disease, non-squamous type, and lymphatic metastasis are associated with high risk of bone metastasis, as well as these risk factors have been elucidated to contribute to poor prognosis.

There are also other prognostic factors which could be used for the prognostic model. Nartthanarung A et al. reported that patients younger than 45 years with bone metastasis at the time of the cervical cancer diagnosis have a poorer prognosis than elderly patients (16). Previous studies also demonstrated that elder cervical cancer patients had adverse prognosis regardless of FIGO stage and histologic subtypes (23, 24). Based on these findings, we developed a predictive score system that can be fabricated to evaluate the probability of the cervical cancer patients with bone metastasis development in the future. These nomograms had better calibration and discriminatory ability, and could be used for clinically meaningful prognostic and predictive assessment of bone metastasis.

Until now, due to the lack of large population-based study with first-diagnosed metastatic cervical cancer, the way of treatment for CCBM patients is still controversial. Hamanishi et al. reported that timely hemipelvectomy for lateral recurrent cervical cancer had reduced tumor pain and prolonged survival (25). Pasricha et al. reported that surgical excision improved the patient's quality of life and palliating pain (26). Park et al. reported that CCBM patients who do not receive therapy for bone metastasis survive for <6 months (22). Hence, for resectable bone metastasis is still far from satisfactory. However, for cervical carcinoma metastatic to the bone, existed evidence demonstrated that concurrent chemotherapy and bisphosphonate administration might be promising (3). Ratanatharathorn et al. reported that radiotherapy provided moderate palliation for treatable patients (27). However, Yu et al. reported that local radiotherapy was merely useful for pain relief, the prognosis was not prolonged (28). Kanayama et al. reported that radiotherapy followed by cisplatin-based chemotherapy for cervical cancer patients with calcaneal metastasis with ideal general condition (29). Collectively, there is no standard treatment option for CCBM patients. With regard to chemotherapy, palliative transcatheter arterial chemoembolization/embolization, compared to intravenous administration, seems to be a suitable treatment method for symptomatic bone metastasis (30). For symptomatic and uncomplicated bone metastasis, a single dose of 8 Gy treatment prescribed to the appropriate target volume is recommended (31). However, a total dose of 30 Gy in 10 fractions is also considered as a standard method with lower rates of pathological fracture and spinal cord compression (32, 33).

In addition, this study inevitably has some limitations. Firstly, due to the insufficient information medical records by SEER database, external validation is warranted in the future. Secondly, the therapeutic experience in our study and many of references are small sized retrospective studies, future large sized and prospective studies are required to provide more instructive information. Thirdly, it was not recommended to perform the survival analysis stratified by radiotherapy and chemotherapy as the records were lacking from the SEER database. Further investigations should be performed to elucidate these results.

## Conclusion

This population-based study depended on the internal validation to evaluate the role of predictive model as to bone metastasis of cervical cancer. In this study, we established seven predictive models for the risk estimation of bone metastasis in CCBM patients. Random forest model performed highest predictive capability among seven predictive models. We also developed a predictive score system based on generalized linear model that can be fabricated to evaluate the probability of the cervical cancer patients with bone metastasis development in the future.

Although we explored seven different machine algorithms to build bone metastasis risk models for cervical cancer patients, there is no significant difference in their predictive performance. In actual clinical practice, we can select multiple models for prediction based on the relevant characteristic information provided by the patient. When the prediction results are consistent, the credibility of the results can be upgraded.

## Data Availability Statement

The original contributions presented in the study are included in the article/supplementary material, further inquiries can be directed to the corresponding author/s.

## Author Contributions

SW and YL designed this study. SZ, JD, and MW drafted the manuscript. BW and JZ prepared all the figures and tables. All authors contributed to the article and approved the submitted version.

## Funding

This work was financially supported by the National Key R&D Program of China (No. 2016YFC1302902) and the Clinical Research Pilot Project of Tongji Hospital, Huazhong University of Science and Technology (No. 2019CR205).

## Conflict of Interest

The authors declare that the research was conducted in the absence of any commercial or financial relationships that could be construed as a potential conflict of interest.

## Publisher's Note

All claims expressed in this article are solely those of the authors and do not necessarily represent those of their affiliated organizations, or those of the publisher, the editors and the reviewers. Any product that may be evaluated in this article, or claim that may be made by its manufacturer, is not guaranteed or endorsed by the publisher.
